# Akira Endo: Father of Statins

**DOI:** 10.7759/cureus.68198

**Published:** 2024-08-30

**Authors:** Michael D Cheshire, Usman A Akbar

**Affiliations:** 1 Internal Medicine/Clinical Lipidology, West Virginia University School of Medicine, Parkersburg, USA; 2 Internal Medicine, West Virginia University School of Medicine, Parkersburg, USA

**Keywords:** atherosclerosis, clinical lipidology, hyperlipidemia, preventive cardiology, hmg-coa reductase inhibitor, statin, lipidology, biography, medical scientist, historical vignette

## Abstract

There was an unprecedented increase in cardiovascular mortality during the 20th century, which ultimately led to a deeper understanding of cholesterol and its role in cardiovascular disease. Akira Endo is a medical innovator who sought to change the tide of this epidemic. He is often referred to as the “Father of Statins” because of his identification of the first 3-hydroxy-3-methylglutaryl coenzyme A (HMG-CoA) inhibitor, which fostered a new era in cholesterol treatment. The offspring of this discovery - statin medications - changed the landscape of cardiovascular prevention and improved outcomes for millions of patients across the globe. Arika Endo used his prior research experience in biochemistry and fungi to create a new way to meet the objective of reducing cardiovascular morbidity and mortality. In this way, he should be recognized not only for this discovery of statins but as a role model for researchers at any stage of their careers.

## Introduction and background

For centuries, researchers, including Leonardo DaVinci, Rudolf Virchow, and others, observed and recorded observations of the atheromatous disease and speculated about its etiology [1]. Pivotal work during the 20th century, however, helped define the metabolism, mechanisms, and effects of cholesterol, including its role in the development of cardiovascular disease. Appreciating the discoveries of this time is important in understanding their influence on Akira Endo’s career. In 1910 Adolf Windaus utilized a new technique for detecting cholesterol and cholesterol esters to examine the association of cholesterol and atheroma formation within arteries, with his studies demonstrating 25 times more cholesterol in arterial walls from atheromatous aortas compared to non-atheromatous aortas [2]. Nikolai Anitschkow continued to investigate this interconnection and in 1913 successfully used rabbit models to demonstrate the association between high-cholesterol diets and the development of atherosclerotic plaque [3]. This work on dietary cholesterol and atherosclerosis was followed by the seven countries study, in which Ancel Keys et al. and Henry Blackburn et al. used large cohort data to establish the association between diet and coronary heart disease (CHD) in human populations [4,5]. Interestingly, information obtained through the seven countries study formed the basis of more contemporary intervention studies on the Mediterranean diet, such as the Lyon Heart Study and PREDIMED [5]. The latter half of the 20th century brought additional cohort data from the Framingham studies, which further identified cholesterol as a significant risk factor for CHD risk and led to its utilization in CHD risk calculators [6-7]. Data from the Framingham cohort studies continued to play a critical role in cardiovascular prevention strategies until well into the 21st century and are still included in some of the more contemporary risk calculators [8-10].

Mortality from cardiovascular disease, mostly attributed to CHD, continued to climb throughout the early to mid-20th century, reaching a peak in 1963 [11]. As a result, diet, lifestyle modification, and lipid-lowering drugs were increasingly used to lower cholesterol levels for the goal of cardiovascular risk reduction [10,12]. Studies of early lipid-lowering therapies demonstrated their usefulness in lipid-lowering and the results of early studies on fibrates, bile acid sequestrants, and niacin are worth noting. The Coronary Drug Project demonstrated the efficacy of niacin in secondary prevention patients, and while it failed to meet its primary mortality endpoint, it met its secondary endpoint of nonfatal myocardial infarction (MI) [13]. Interestingly, niacin-treated patients later demonstrated significant mortality reduction at 15-year follow-up [14]. The Lipid Research Clinics Study, which evaluated the use of cholestyramine in high-risk patients, showed a 19% reduction in the risk of the primary end point of death from CHD and/or nonfatal MI [15]. Last, the Helsinki Heart Study compared gemfibrozil versus placebo in primary prevention patients with hyperlipidemia, meeting its primary endpoint of fatal and non-fatal MI and cardiac death and showing a 34% reduction in the cumulative rate of cardiac endpoints at five years [16]. While all these drugs demonstrated some cardiovascular benefit, they were limited mostly by side effects and even though we had medications to lower cholesterol, more potent and better tolerated pharmacologic therapies were needed.

A critical discovery leading to the development of new lipid-lowering treatments was the elucidation of the mechanism of cholesterol synthesis by Dr. Konrad Bloch, leading to a share of the Nobel Prize in Physiology or Medicine in 1964 [17]. Soon after, in 1973, Michael S. Brown and Joseph L. Goldstein further contributed to the understanding of cholesterol regulation by identifying the LDL receptor, a discovery that led to their receiving a Nobel Prize in Physiology or Medicine in 1985 [18].

**Figure 1:**
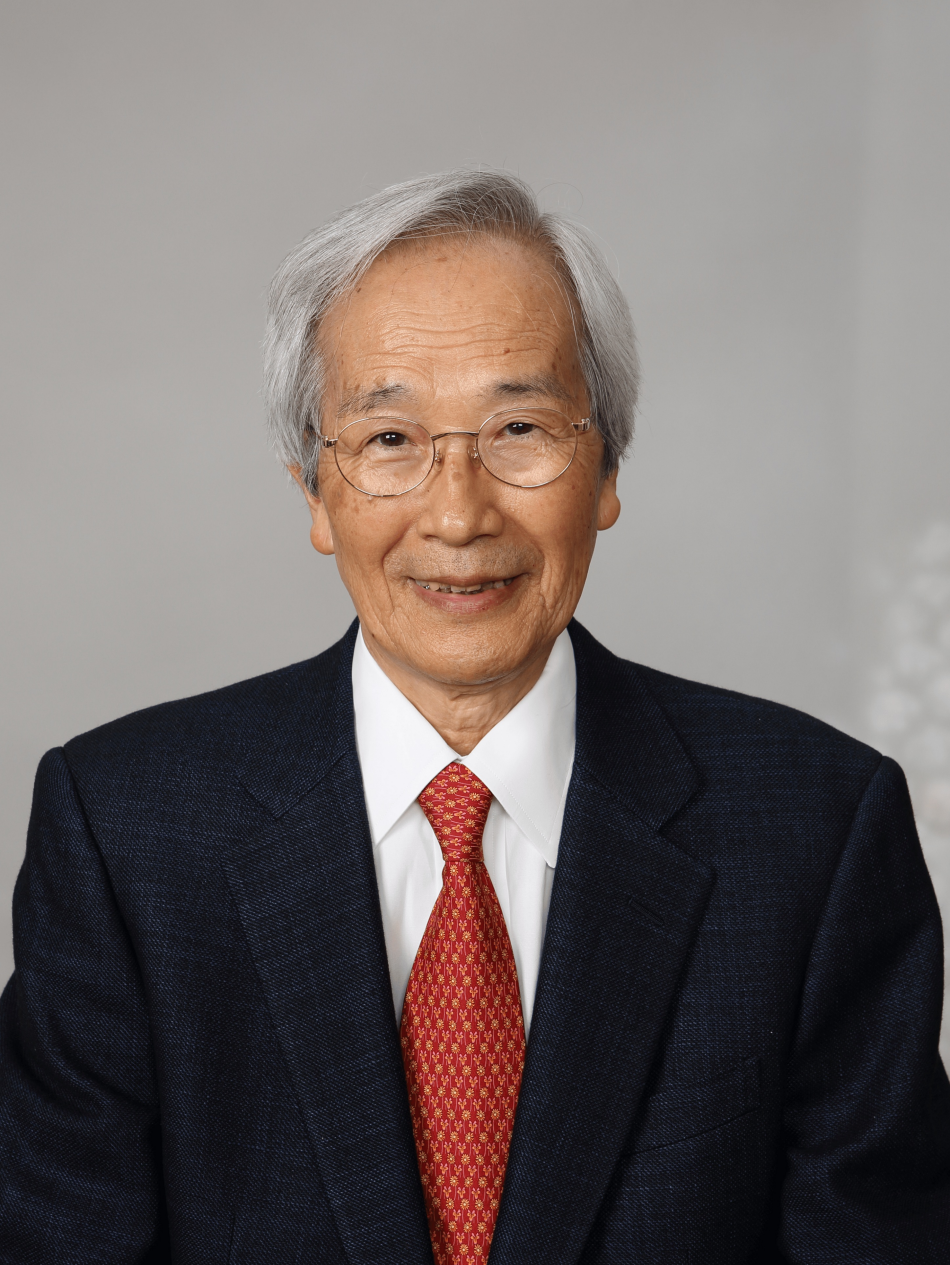
Akira Endo Akira Endo at the time of receiving the Gairdner International Award in 2017 [19]. Permission to publish this photograph has been obtained from The Gairdner Foundation.

The discoveries set the stage for Akira Endo’s research. This article examines his work and contributions, specifically the isolation of a 3-hydroxy-3-methylglutaryl coenzyme A (HMG-CoA) reductase inhibitor with the intent of developing a new pharmacologic option for reducing serum cholesterol and improving cardiovascular outcomes. Figure 1 shows a photograph of Akira Endo taken in 2017.

## Review

Early life and influences

Ralph Waldo Emerson said, “Do not go where the path may lead, go instead where there is no path and leave a trail.” These are inspiring words for any researcher, and Akira Endo provides a wonderful example of their fulfillment. Born in November 1933 in the rural farming community of Hagishiyuri (now known as Yurihonjo), Akira Endo’s circumstances did not present a clear path to the career and discoveries he would achieve in his lifetime [20]. Yet the trail he blazed led to the identification of a drug class that has benefited millions of people across the globe.

His earliest influence was his grandfather, who imparted his own interest in science and medicine to young Akira Endo, so much so that even at the age of eight, he dreamed of becoming a scientist [21]. Two other influences affected the direction he would take in his education. These include the work of Alexander Fleming, whose discovery of penicillin saved countless lives, and the work of Hideyo Noguchi, a Japanese scientist who traveled to the United States to conduct studies on syphilis and yellow fever [21]. Inspired by these researchers, he developed his academic plan. After completing high school in Akita, Akira Endo studied at Tohoku University’s College of Agriculture in Sendai where he concentrated on organic chemistry, biochemistry, and applied microbiology before beginning work at Sankyo Pharmaceuticals in Tokyo in 1957 [21,22].

It was at this time that Dr. Konrad Bloch may have had the greatest direct influence on Akira Endo’s later discoveries. Dr. Bloch was a biochemist, who began studying cholesterol metabolism in the 1940s and received a half share of the 1964 Nobel Prize in Physiology or Medicine for his work on describing the mechanism of cholesterol synthesis [23]. His research was instrumental in elucidating the biochemical pathways involved in cholesterol synthesis, particularly identifying the importance of HMG-CoA reductase, the enzyme responsible for catalyzing the conversion of HMG-CoA to mevalonate a critical and rate-limiting step in cholesterol biosynthesis. Dr. Bloch and his colleagues used radiolabeled precursors, such as acetate, to trace the incorporation of carbon atoms into cholesterol in living organisms, systematically analyzing the intermediates formed during the process [24]. This meticulous approach allowed them to pinpoint specific steps and enzymes within the pathway. The identification of HMG-CoA reductase as the enzyme catalyzing this key early step was further confirmed by isolating the enzyme and demonstrating its activity in vitro [25]. Dr. Bloch’s work also revealed the regulatory mechanisms that modulate HMG-CoA reductase activity based on cellular cholesterol levels, establishing it as a crucial control point in cholesterol synthesis. This discovery laid the groundwork for later therapeutic interventions targeting this enzyme, which became the basis for developing statins-revolutionary drugs in the management of hypercholesterolemia and cardiovascular disease​ [26].

In 1965, Akira Endo wrote to Dr. Bloch inquiring about post-doctoral research opportunities in his laboratory at Harvard University, but the positions were no longer available [24]. By fortune or fate, Akira Endo began the study of phospholipids at the Albert Einstein College of Medicine in New York in 1966 and received mentorship on the role hypercholesterolemia plays as a major risk factor for CHD [27]. His time in New York allowed him to observe the contrast in dietary habits between the United States and Japan, the prevalence of overweight individuals, and the frequency of ambulances transporting heart attack victims to local hospitals [22]. These experiences inspired a new sense of purpose and research focus.

Discovery and contributions

Akira Endo returned to Sankyo in 1968, and in 1970 he solidified a hypothesis that would lead to one of his greatest discoveries. He hypothesized that inhibition of HMG-CoA reductase would lower plasma cholesterol and postulated that this mechanism of inhibition inherently existed within microbes as a possible system of defense against other microbes that utilized associated compounds for growth [24].

In 1971, Akira Endo, alongside Dr. Masao Kuroda, initiated a groundbreaking project aimed at identifying microbial metabolites that could inhibit HMG-CoA reductase, the key enzyme in cholesterol biosynthesis. They hypothesized that inhibiting this enzyme would reduce plasma cholesterol levels in humans, lowering the risk of CHD [22]. Their search led to the isolation of a potent HMG-CoA reductase inhibitor, mevastatin (initially called ML-236B or compactin), from the mold “*Penicillium citrinum*.” This discovery was significant as mevastatin demonstrated a substantial ability to lower LDL-cholesterol levels in both experimental animals and humans with primary hypercholesterolemia. By the end of 1973, they had determined the structure of mevastatin using spectroscopic, chemical, and X-ray crystallographic methods [27].

The mechanism of mevastatin involves the inhibition of sterol synthesis by blocking HMG-CoA reductase, thereby reducing the conversion of HMG-CoA to mevalonate, a key precursor in cholesterol biosynthesis. This inhibition was competitive and reversible, with mevastatin exhibiting a high affinity for the enzyme [24].

**Figure 2:**
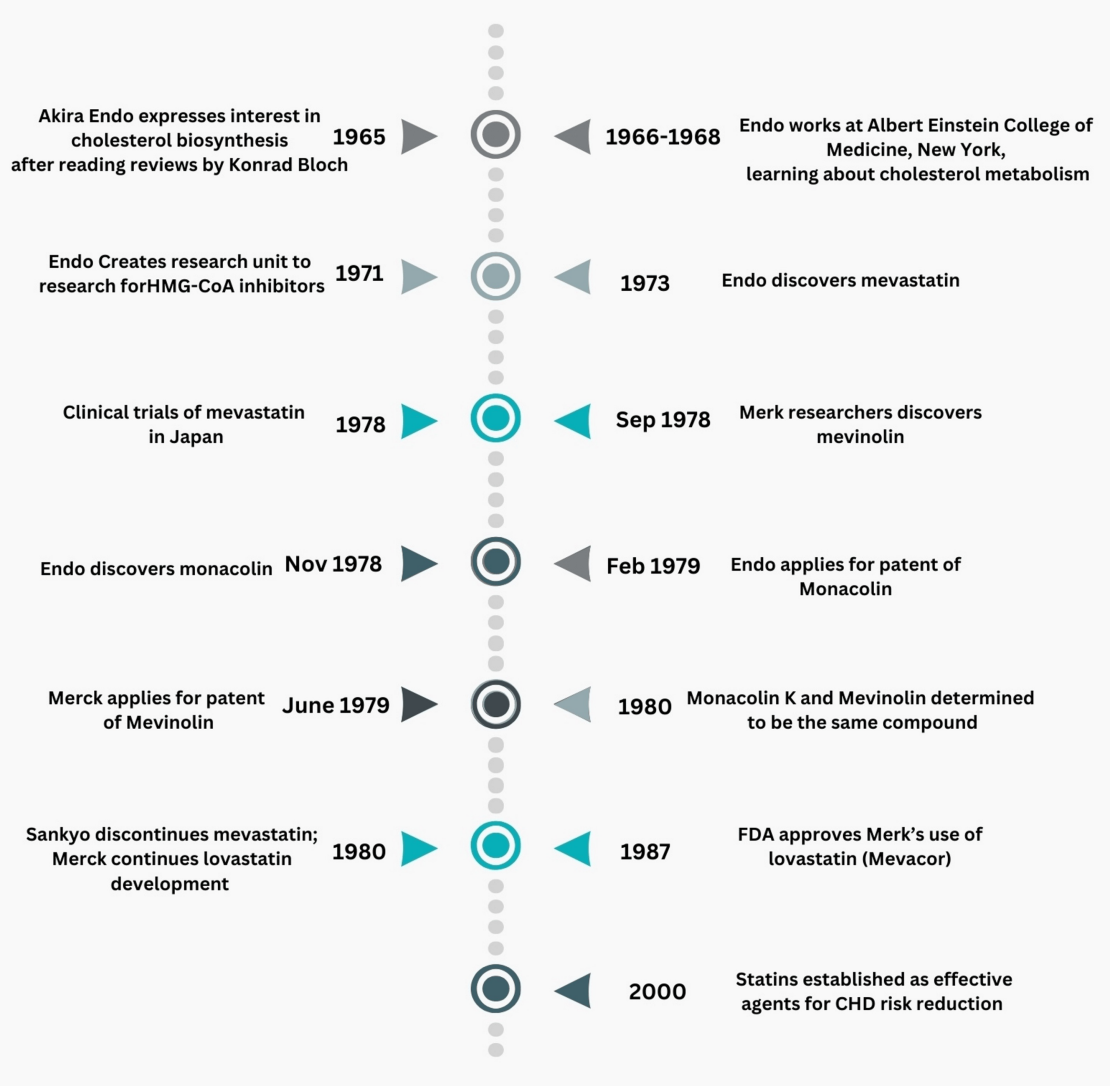
Timeline of Akira Endo's work This timeline displays important dates regarding Akira Endo's role in the discovery and commercialization of statin medications [22,24].

In subsequent studies, mevastatin significantly reduced LDL-cholesterol levels in various animal models, including hens, dogs, and monkeys [28]. Notably, in 1978, clinical trials in Japan showed that mevastatin could lower plasma total and LDL cholesterol by 20-40% at doses of 15-60 mg/day without serious side effects [29]. Following the success of mevastatin, Akira Endo left Sankyo and joined Tokyo Noko University, where he isolated three mevastatin analogues - monacolin J, monacolin K, and monacolin L - from “*Monascus ruber*” [22,24]. Of these, monacolin K (later known as lovastatin) was discovered in November 1978 and proved to be slightly more effective than mevastatin in inhibiting HMG-CoA reductase [22]. Akira Endo applied for a patent for monacolin K in 30 countries in February 1979 [24]. Meanwhile, Alfred Alberts, Carl Hoffman, and associates - all researchers at Merck Sharp and Dohme Research Laboratories - had been diligently searching for an HMG-CoA inhibitor of their own, isolating mevinolin (later called lovastatin) from *Aspergillus terreus* in September 1978 and applying for a patent in June 1979 [24]. It was later determined that mevinolin and monocolin K were the same compound (lovastatin) [24]. Given this timeline (as seen in Figure 2), researchers on the other side of the globe discovered lovastatin just three months ahead of Akira Endo! Interestingly, Merck’s patent for mevinolin came four months after that of monocolin K [24]. Subsequently, the patent rights for monocolin K were transferred from Tokyo Noko University to Sankyo [24]. Lovastatin was developed by Merck and underwent extensive testing, ultimately being approved for clinical use by the FDA in 1987 under the name Mevacor [22]. Even though Sankyo did not pursue commercialization of monocolin K, the company generated money through the licensing of this compound based on whether countries recognized “time of application” over “time of invention” [24].

Akira Endo's discovery of mevastatin and the subsequent development of lovastatin paved the way for the creation of several other statin drugs, including simvastatin, pravastatin, fluvastatin, and atorvastatin. These drugs have been instrumental in reducing cardiovascular morbidity and mortality by effectively lowering cholesterol levels in millions of patients worldwide [2]. Endo's work exemplifies the power of applying interdisciplinary knowledge and persistence in research. His contributions to the development of statins have not only transformed the treatment of hypercholesterolemia but also provided a model for future drug discovery and development.

As we began writing this manuscript, Akira Endo was still living. Sadly, he died on June 5, 2024, before it was completed [30]. We are humbled and honored to commemorate him through this work.

## Conclusions

Akira Endo was professionally and personally impacted by his time in New York and felt compelled to apply his knowledge of biochemistry and research experience with fungi to reduce the burden of CVD. His background in microbiology and work with fungi made him particularly prepared to identify an HMG-CoA reductase inhibitor from fungi. His work provides a shining example of applying past experiences to solve unfamiliar problems. His benevolent work in developing such an influential class of pharmaceuticals should be recognized and appreciated by clinicians, researchers, and patients.
